# CDCA2 promotes lung adenocarcinoma cell proliferation and predicts poor survival in lung adenocarcinoma patients

**DOI:** 10.18632/oncotarget.15519

**Published:** 2017-02-19

**Authors:** Run Shi, Chunrong Zhang, Yaqin Wu, Xin Wang, Qi Sun, Jing Sun, Wenjie Xia, Gaochao Dong, Anpeng Wang, Feng Jiang, Lin Xu

**Affiliations:** ^1^ Jiangsu Key Laboratory of Molecular and Translational Cancer Research, Cancer Institute of Jiangsu Province, Jiangsu, China; ^2^ Department of Thoracic Surgery, Affiliated Cancer Hospital, Nanjing Medical University, Jiangsu, China; ^3^ The First Clinical College of Nanjing Medical University, Nanjing, China; ^4^ The Fourth Clinical College of Nanjing Medical University, Nanjing, China; ^5^ Department of Cardiothoracic Surgery at Jinling Hospital, Southern Medical University, Nanjing, China; ^6^ Department of Thoracic Surgery, Nantong Tumor Hospital, Jiangsu, China

**Keywords:** CDCA2, lung adenocarcinoma, TCGA, proliferation, prognosis

## Abstract

Cell division cycle associated 2(CDCA2) is overexpressed in neuroblastoma and oral squamous cell carcinoma, and its overexpression positively correlates to tumor progression. However, the biological and clinical significance of CDCA2 in lung adenocarcinoma(LAC) has never been investigated. We determined the expression profile and clinical significance of CDCA2 using The Cancer Genome Atlas(TCGA) and tissue microarray(TMA). Furthermore, we explored the biological function of CDCA2 both *in vitro* and *in vivo*. A great upregulation of CDCA2 was observed in LAC tissues compared with adjacent normal tissues. Importantly, Cox regression analysis indicated that high level of CDCA2 was an independent risk factor for overall survival(OS) in LAC patients (TCGA: HR = 1.720, *p* = 0.004; TMA: HR = 1.971, *p* = 0.023). Inhibition of CDCA2 suppressed the proliferation of LAC cells via G1 phase arrest by downregulating cyclin E1(CCNE1), while overexpression of CDCA2 promoted LAC cells proliferation by upregulating CCNE1. Moreover, the oncogenic activity of CDCA2 was also confirmed *in vivo*. In conclusion, CDCA2 promotes proliferation of LAC cells and predicts poor prognosis in LAC patients. CDCA2 might play a significant role in LAC progression.

## INTRODUCTION

Lung cancer is the most common malignancy and the leading cause of cancer-related death worldwide [1]. Two main histological types are included: non-small cell lung cancer(NSCLC) and small cell lung cancer. NSCLC constitutes about 85% of all lung cancers, and lung adenocarcinoma(LAC) has been the most common subtype of NSCLC in recent years [1, 2]. Despite advances in lung cancer therapies, prognosis of NSCLC is still unfavorable, with an overall 5-year survival rate less than 15% [3]. Therefore, further investigation on identification of prognostic biomarkers and potential drug targets is eagerly needed to provide better prognosis and individualized treatment.

Cell division cycle associated 2(CDCA2), a nuclear protein that binds to protein phosphatase 1 γ(PP1γ), regulates the DNA damage response(DDR) in cell cycle [4, 5]. CDCA2-PP1γ complex cooperates with condensin to preserve the characteristic chromosome architecture during mitosis [6], and the complex is an anaphase-activated protein phosphatase that is regulated via CDCA2 phosphorylation [7]. Moreover, CDCA2 promotes de-phosphorylation of the major mitotic histone H3 in a PP1-dependent manner [8]. Though CDCA2 is overexpressed in aggressive neuroblastoma [9] and oral squamous cell carcinoma [10], the expression profile and biological function of CDCA2 in NSCLC still remain unknown.

In this study, for the first time we have shown aberrant expression and clinical significance of CDCA2 in LAC. In addition, we also show biological function of CDCA2 *in vitro* and *in vivo*. Our findings suggest that CDCA2 might play a significant role in LAC progression.

## RESULTS

### CDCA2 is upregulated in LAC and correlates with more aggressive clinical characteristics in TCGA dataset

**Figure 1:**
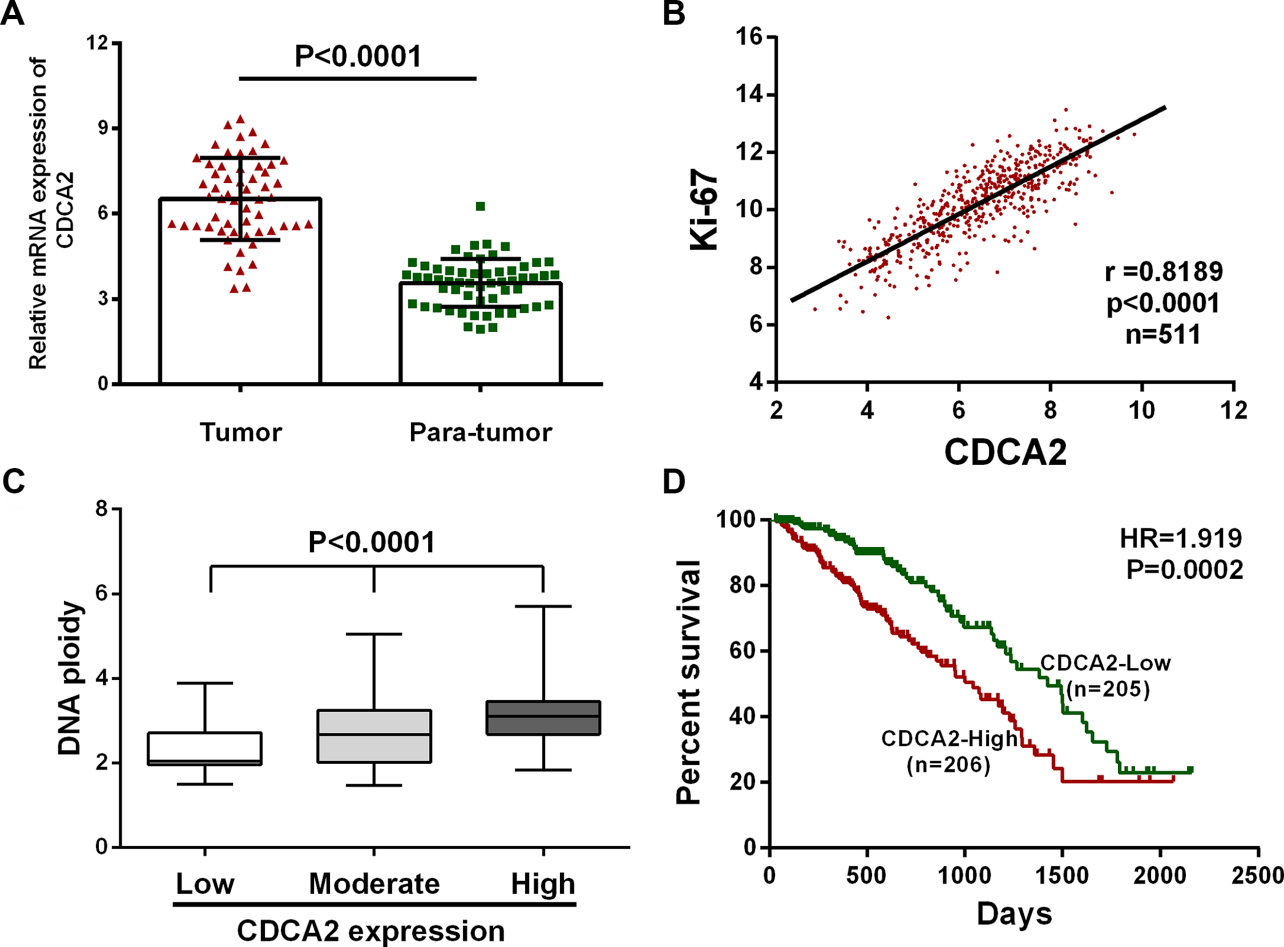
CDCA2 is upregulated in LAC tissues and correlates with more aggressive clinical characteristics in TCGA dataset (**A**) CDCA2 is over-expressed in 94.7% (54 of 57) of LAC tissues compared with para-tumor normal tissues. (**B** and **C**) CDCA2 was found to be positively correlated with Ki-67 (*r* = 0.8189, *p* < 0.0001) and DNA ploidy (*p* < 0.0001) in LAC tissues. (**D**) Kaplan-Meier survival analysis indicated that higher CDCA2 expression is associated with worse overall survival in LAC patients (HR = 1.919, *p* = 0.0002).

By analyzing *TCGA_LUAD_exp_HiSeqV2-2015-02-24* dataset, we found that mRNA expression of CDCA2 was upregulated in 54 (94.7%) LAC tissues out of the 57 paired tissues (the tumor and the paired normal lung tissue from a same patient) (Figure 1A). Moreover, CDCA2 expression was positively correlated with Ki-67 expression (*r* = 0.8189, *p* < 0.0001) and DNA ploidy (*p* < 0.0001) in the 511 LAC tissues in TCGA dataset (Figure 1B and 1C). Then, 473 LAC patients with full-scale clinical information were extracted for further analysis (Details shown in Supplementary Table 1). We designated the median expression value as a cutoff point, and the 473 LAC patients were divided into two groups: CDCA2-low group (*n* = 236) and CDCA2-high group (*n* = 237). As shown in Table 1, chi-square test revealed that CDCA2 mRNA expression was significantly correlated with sex (*p* < 0.0001), primary tumor size (*p* = 0.0003) and TNM stage (*p* = 0.0024).

**Table 1:** Correlation between CDCA2 expression and clinical characteristics in TCGA LAC dataset (*n* = 473)

Characteristics	CDCA2-low cases	CDCA2-high cases	*P* value
Age at diagnosis(years)			0.3938
≤ 60	72	81	
> 60	164	156	
Sex			< 0.0001*
Male	85	135	
Female	151	102	
Primary tumor size			0.0003*
T1	101	61	
T2	105	144	
T3–4	30	32	
Lymph node status			0.0881
N0	163	146	
N1–3	73	91	
Tumor stage			0.0024*
I	139	115	
II	62	56	
III–IV	35	66	

* Significant correlation.

After excluding patients without follow-up, 411 patients remained (Details shown in Supplementary Table 2). Overall survival curves were plotted, and Cox regression analysis was performed to evaluate the prognostic value of CDCA2 in LAC. Compared with CDCA2-low group (*n* = 205), CDCA2-high group (*n* = 206) displayed poor OS (HR = 1.919, *p* = 0.0002) (Figure 1D). As shown in Table 2, multivariate Cox regression analysis further revealed that high level of CDCA2 mRNA expression was an independent risk factor for OS in LAC patients (HR = 1.720, 95% CI = 1.189–2.489, *p* = 0.004).

**Table 2:** Cox regression analysis of overall survival in LAC patients in TCGA LAC dataset

	Univariate analysis			Multivariate analysis		
	HR	*P* value	95% CI	HR	*P* value	95% CI
Gender (Male vs Female)	1.125	0.498	0.800–1.581			
Age (> 60 years vs ≤ 60 years)	1.267	0.211	0.874–1.835			
Primary tumor size(T_2–4_ vs T_1_)	1.745	0.008*	1.155–2.637	1.169	0.506	0.738–1.851
Lymph node status(N_1–3_ vs N_0_)	2.93	< 0.001*	2.066–4.157	1.287	0.392	0.723–2.292
TNM stage(Stag II–IV vs Stage I)	3.229	< 0.001*	2.226–4.684	2.143	0.018*	1.142–4.021
CDCA2 expression(High vs Low)	1.786	< 0.001*	1.251–2.550	1.720	0.004*	1.189–2.489
Ki-67 expression(High vs Low)	1.712	0.002*	1.218–2.408	1.402	0.200	0.837–2.349

* Significant correlation,

Features with *p* value < 0.05 in univariate analysis were taken into multivariate analysis.

### High level of CDCA2 protein predicts worse prognosis in LAC patients

**Figure 2:**
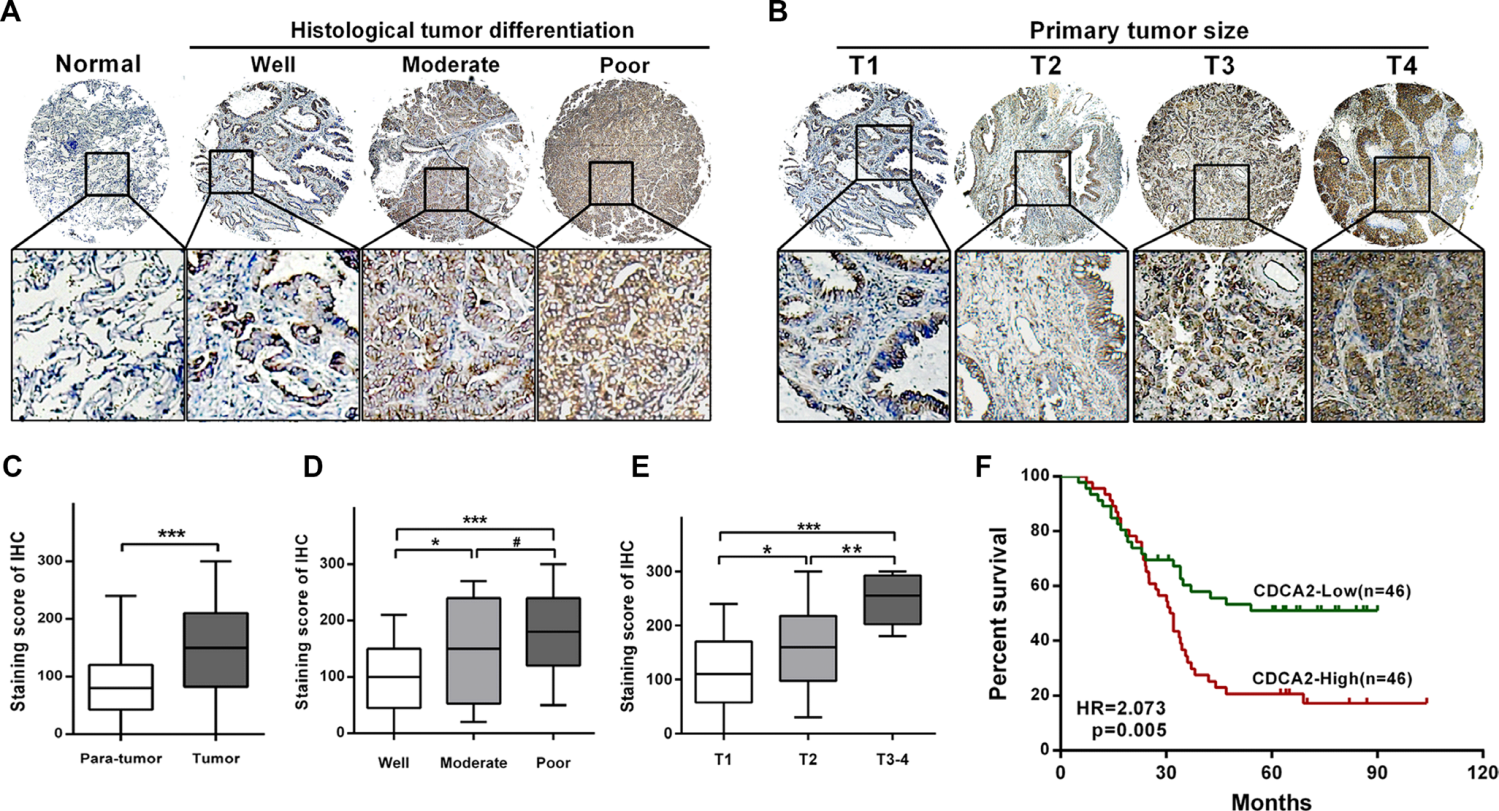
Tissue microarray analysis (**A**) Representative images of CDCA2 staining in different histological differentiation levels (from well to poor). (**B**) Representative images of CDCA2 staining in different tumor sizes (from T1 to T4). (**C**) CDCA2 staining score was significantly increased in LAC tissues compared to adjacent normal tissues. (**D**) CDCA2 staining score was significantly increased along with worse differentiation. (**E**) CDCA2 staining score was significantly increased along with advanced T stage. (**F**) Kaplan-Meier survival analysis and log-rank test indicated that high level of CDCA2 expression was associated with poor overall survival (HR = 2.073, *p* = 0.005). *p < 0.05, **p < 0.01, ***p < 0.001, ^#^No significance.

To further evaluate the clinical utility of CDCA2 in the prognosis of LAC patients, we applied our own LAC tissue microarray containing 92 pairs of LAC and matched non-tumor tissues with long time follow-up records [11]. The result showed that the score of CDCA2 staining was significantly increased in LAC tissues compared with adjacent normal tissues (Figure 2A and 2C). Moreover, the score of CDCA2 staining was significantly increased along with worse differentiation (Figure 2A and 2D) and advanced T stage (Figure 2B and 2E) in LAC tissues. Then we designated the median IHC staining score as a cutoff value, and the 92 LAC patients were divided into two groups: low-CDCA2 expression group (*n* = 46) and high-CDCA2 expression group (*n* = 46). Consistent with findings in TCGA, chi-square test also revealed that CDCA2 expression positively correlated with differentiation (*P* = 0.020), primary tumor size (*P* = 0.0025) and TNM stage (*P* = 0.0171) (Table 3). OS was calculated by Kaplan-Meier analysis and log-rank test. As shown in Figure 2F, patients with higher CDCA2 expression exhibited worse OS (HR = 2.073, *P* = 0.005). The multivariate analysis revealed that high level of CDCA2 was an independent risk factor for OS in LAC patients. The group with higher expression of CDCA2 exhibited shorter OS rate (HR = 1.971, 95% CI = 1.100–3.533, *p* = 0.023) (Table 4).

**Table 3:** Correlation between CDCA2 expression and clinical characteristics in TMA(*n* = 92)

Characteristics	CDCA2-low cases	CDCA2-high cases	*P* value
Age at diagnosis(years)			0.1420
≤ 60	22	29	
> 60	24	17	
Gender			0.2413
Male	36	31	
Female	10	15	
Differentiation			0.0220*
Well	17	8	
Moderate	12	8	
Poor	17	30	
Primary tumor size			0.0025*
T1	24	10	
T2–4	22	36	
Lymph node status			0.0940
N0	29	21	
N1–3	17	25	
Tumor stage			0.0171*
I	20	14	
II	16	9	
III	10	23	

* Significant correlation.

**Table 4:** Cox regression analysis of overall survival in LAC patients in TMA

	Univariate analysis			Multivariate analysis		
	HR	*P* value	95% CI	HR	*P* value	95% CI
Gender (Male vs Female)	0.952	0.866	0.536–1.690			
Age (> 60 years vs ≤ 60 years)	1.982	0.009*	1.183–3.320	2.013	0.026*	1.089–3.724
Differentiation (Poor vs Moderate & Well)	2.594	0.001*	1.469–4.579			
Primary tumor size (T_2–4_ vs T_1_)	1.106	0.710	0.649–1.887			
Lymph node status (N_1–3_ vs N_0_)	2.289	0.002*	1.364–3.839	1.585	0.139	0.861–2.919
TNM stage (Stage II–IV vs Stage I)	2.365	0.001*	1.413–3.958	2.064	0.025*	1.096–3.888
CDCA2 expression (High vs Low)	2.108	0.006*	1.236–3.594	1.971	0.023*	1.100–3.533

* Significant correlation.

Features with *p* value < 0.05 in univariate analysis were taken into multivariate analysis.

### Knockdown of CDCA2 inhibits LAC cells proliferation via inducing G1 phase arrest

**Figure 3:**
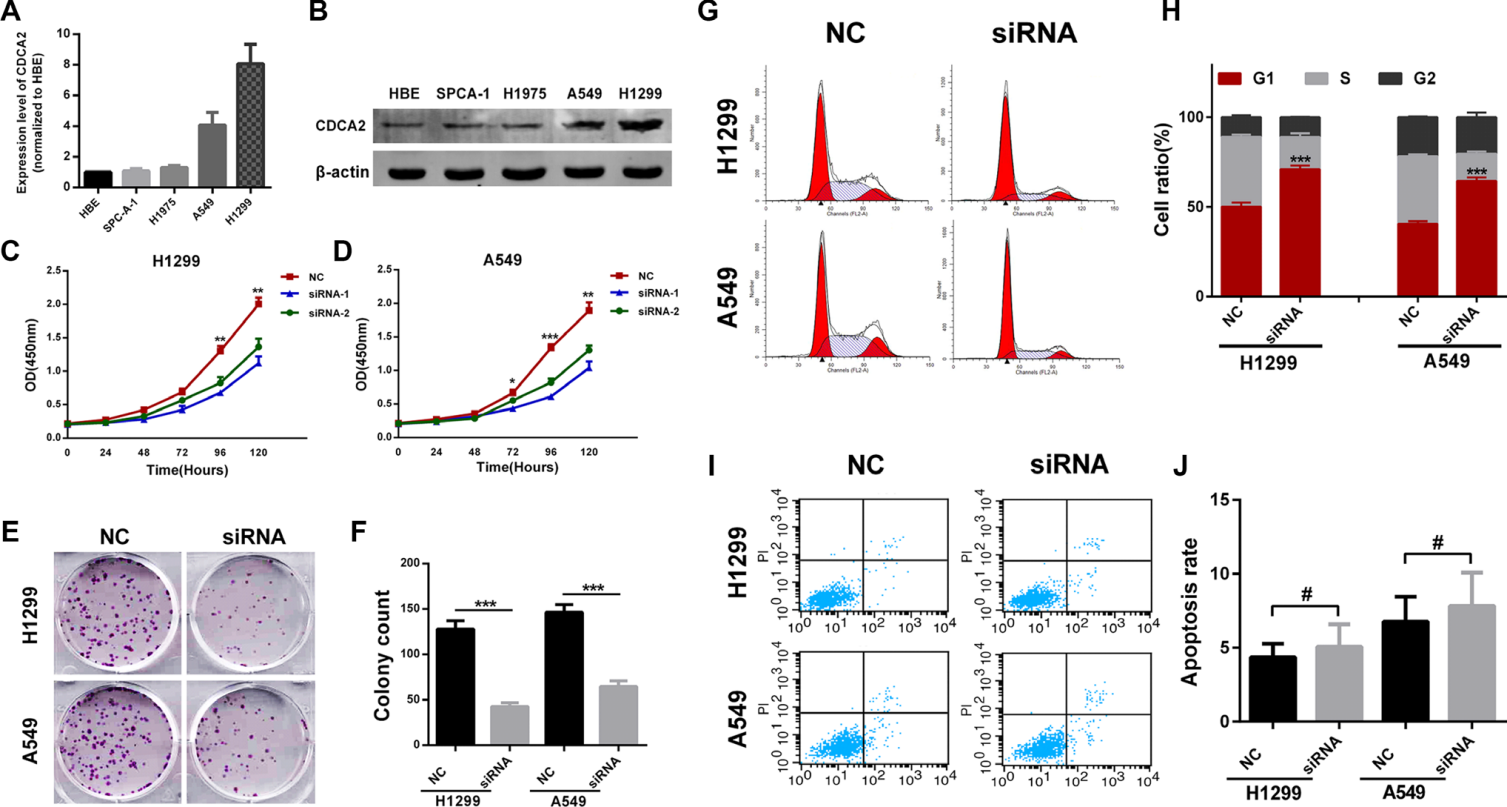
Knockdown of CDCA2 inhibited LAC cell lines proliferation and induced G1 phase arrest *in vitro* (**A** and **B**) A549 and H1299 cell lines were chosen as appropriate cellular models to knockdown CDCA2 for further investigation. (**C** and **D**) CCK-8 assays showed knockdown of CDCA2 inhibited both A549 and H1299 cells proliferation. (**E** and **F**) Colony numbers of A549 and H1299 cells transfected with siRNA-CDCA2 were less than those transfected with siRNA-NC. (**G** and **H**) H1299 and A549 cells transfected with siRNA-CDCA2 exhibited more arrest at G1 phase than those transfected with siRNA-NC. (**I** and **J**) No difference of apoptosis was observed between siRNA-CDCA2 group and siRNA-NC group in H1299 and A549 cells. *p < 0.05, **p < 0.01, ***p < 0.001, ^#^No significance.

In order to choose appropriate cellular models for further investigation, we compared the expression level of CDCA2 in different LAC cell lines. CDCA2 was markedly upregulated in H1299 and A549 cell lines when compared with normal human bronchial epithelial(HBE) cells using qRT-PCR and western blot (Figure 3A and 3B). To investigate the biological function of CDCA2 *in vitro*, two different effective siRNAs were used to knockdown CDCA2.

As shown in Figure 3C and 3D, cell-counting kit 8 (CCK-8) assays revealed that knockdown of CDCA2 significantly inhibited proliferation of both H1299 and A549 cells. Moreover, si-CDCA2 transfected groups had significantly fewer colonies than si-NC groups (Figure 3E and 3F). Lastly, the effects of CDCA2 on cell cycle distribution and apoptosis were assessed by flow cytometry analysis. As shown in Figure 3G and 3H, si-CDCA2 treatment induced increased percentage H1299 and A549 cells in G1 phase compared to si-NC group. However, no difference of apoptosis was observed between the two groups (Figure 3I and 3J).

**Figure 4:**
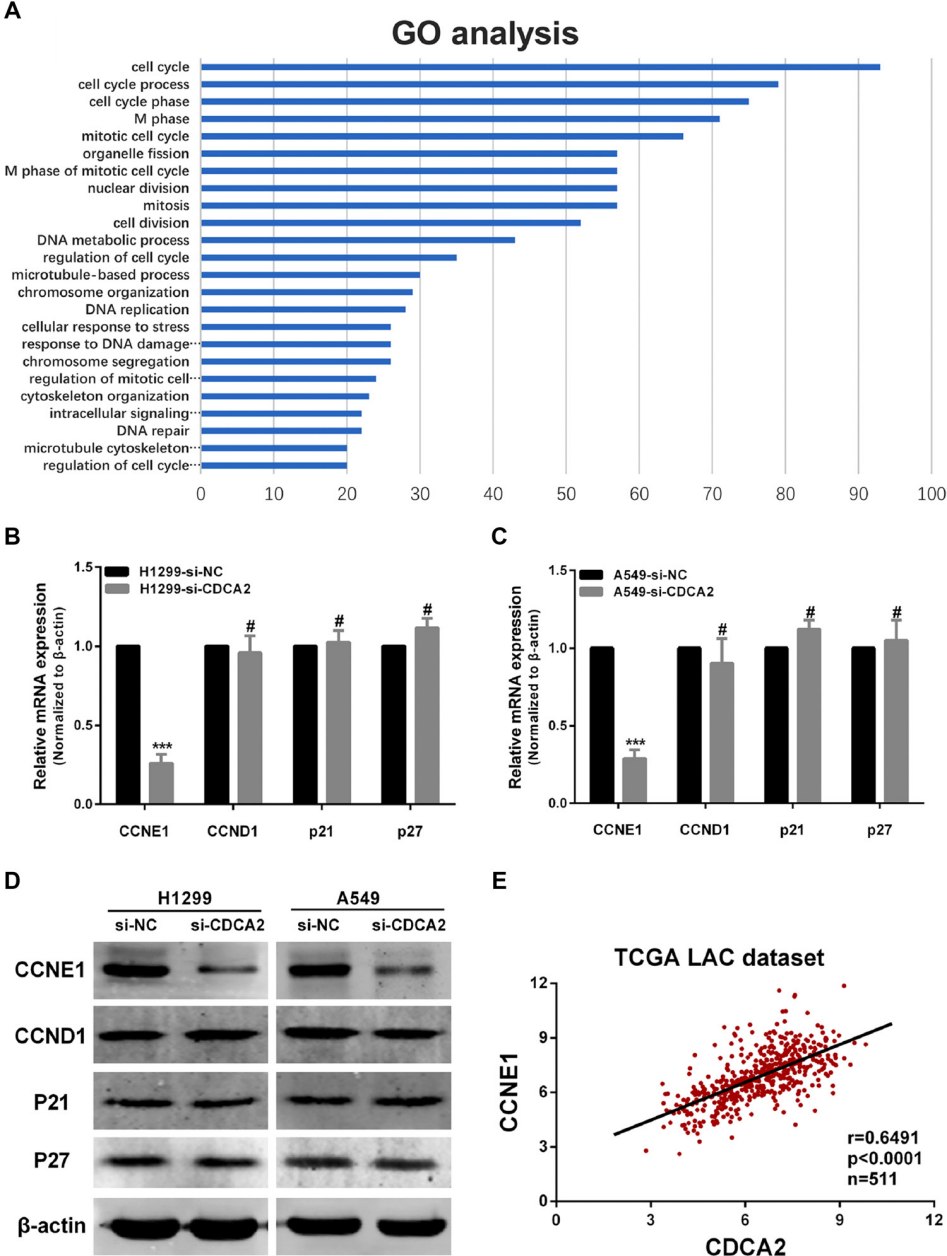
Knockdown of CDCA2 influences CCNE1 expression (**A**) Genes co-expressed with CDCA2 are enriched in the “cell cycle” pathway using GO enrichment analysis. (**B** and **C**) qRT-PCR showed that CCNE1 mRNA levels decreased after siRNA-CDCA2 transfection in both H1299 and A549 cells, while CCND1, p21 or p27 was not significantly altered. (**D**) Western bolt also showed that CCNE1 protein levels decreased after siRNA-CDCA2 transfection in both H1299 and A549 cells, while CCND1, p21 or p27 was not significantly altered. (**E**) Pearson test showed that CDCA2 positively correlated with CCNE1 (*r* = 0.6491, *p* < 0.0001, *n* = 511) in LAC tissues in TCGA dataset. ***p < 0.001, ^#^No significance.

We next used Gene Ontology (GO) enrichment analysis on a list of 160 genes which are highly correlated with CDCA2 in TCGA LAC dataset. Most of the genes were enriched in the “cell cycle” pathway (Figure 4A). Considering that knockdown of CDCA2 induces G1 phase arrest *in vitro*, we sought to determine whether the expression levels of certain critical G1 phase genes or G1/S transition regulators, including CCND1, CCNE1, p21 and p27, were altered in si-CDCA2 treated cells. Compared with si-NC transfected cells, qRT-PCR and western blot showed that both mRNA and protein expression levels of CCNE1 were significantly decreased in si-CDCA2 transfected H1299 and A549 cells compared to NC groups. In contrast, expression of CCND1, p21 or p27 showed no significant difference in two groups (Figure 4B, 4C and 4D). Then we performed correlation analysis between CDCA2 and CCNE1, CCND1, p21 and p27 in TCGA LAC database, respectively. Pearson test revealed that CCNE1 expression was positively correlated with CDCA2(*r* = 0.6491, *p* < 0.0001) (Figure 4E), but CCND1, p21 and p27 were not (Supplementary Figure 1).

### Overexpression of CDCA2 promotes LAC cells proliferation by upregulating CCNE1

**Figure 5:**
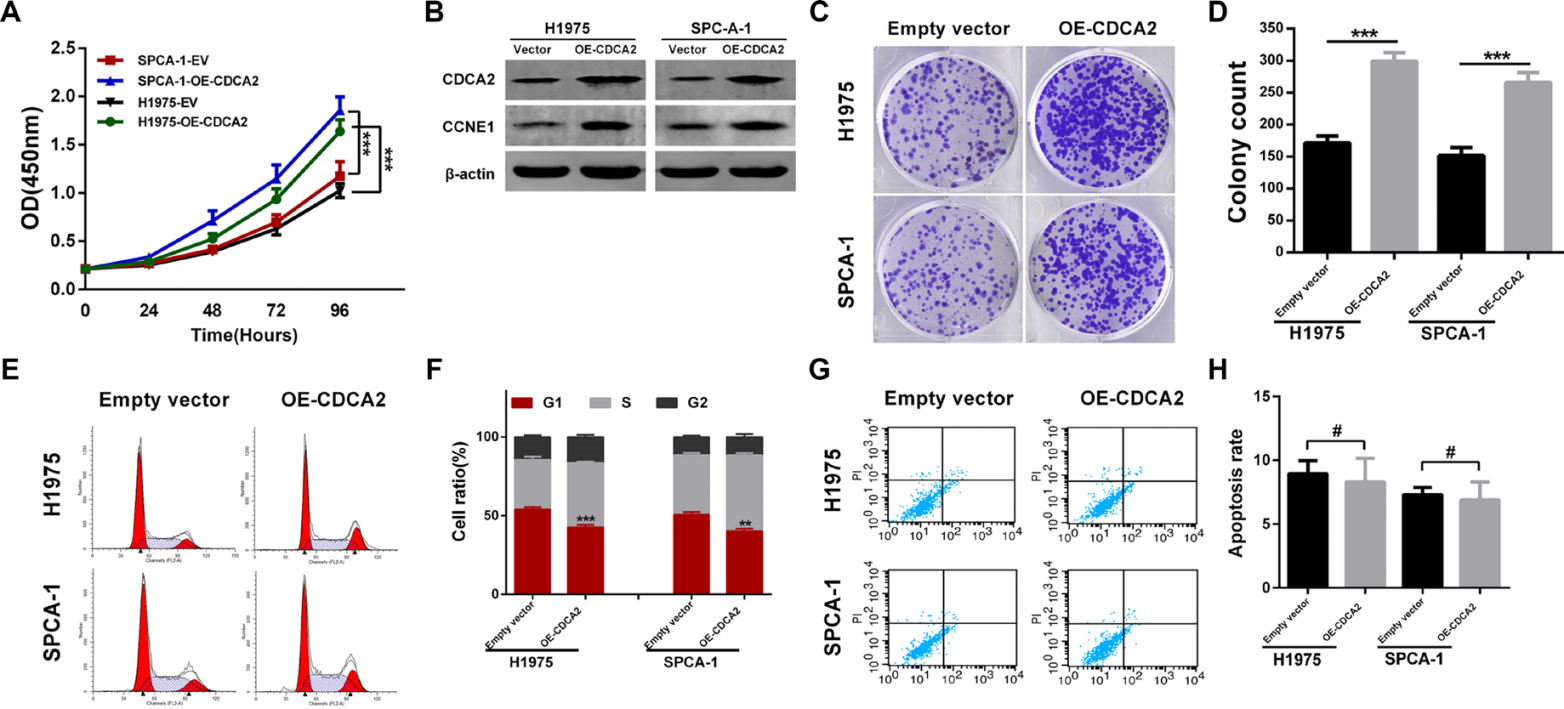
Overexpression of CDCA2 enhanced LAC cells proliferative ability *in vitro* (**A**) CCK-8 assays showed that overexpression of CDCA2 promoted H1975 and SPCA-1 cells proliferation compared to empty vector (EV) control. (**B**) Transfection efficiency of CDCA2 overexpression was measured using western blot. CCNE1 was increased after CDCA2 expression was upregulated. (**C** and **D**) Colony numbers of H1975 and SPCA-1 cells transfected with CDCA2 plasmid were significantly more than those in EV groups. (**E** and **F**) H1975 and SPCA-1 cells transfected with CDCA2 plasmid exhibited less percentage at G1 phase than those in EV groups. (**G** and **H**) No difference of apoptosis was observed between CDCA2-overexpression group and EV group in H1975 and SPCA-1 cells. **p < 0.01, ***p < 0.001, ^#^No significance.

To further evaluate the biological function of CDCA2 in LAC, we examined the effect of CDCA2 overexpression on proliferation of H1975 and SPCA-1 cells because of their relative lower expression (Figure 3A and 3B). These cells were transfected with either CDCA2-overexpression plasmid or empty vector(EV). As shown in Figure 5A, CCK-8 assays revealed that the proliferation abilities of H1975 and SPCA-1 cells overexpressing CDCA2 were significantly enhanced compared to those of vector control cells. Western blot showed that CCNE1 levels were also increased in CDCA2-overexpression groups (Figure 5B). Colony formation assay showed that the CDCA2-overexpression groups had significantly more colony numbers than vector control groups (Figure 5C and 5D). Lastly, the effects of overexpression of CDCA2 on cell cycle distribution and apoptosis were assessed by flow cytometry analysis. As shown in Figure 5E and 5F, more percentage of G1-phase cells were observed in vector control groups compared with CDCA2-overexpression groups. In contrast, no difference of apoptosis was observed between vector control and CDCA2-overexpression groups (Figure 5G and 5H).

### Enforced overexpression of CCNE1 partly rescues the malignant phenotypes in CDCA2-knockdown cells

**Figure 6:**
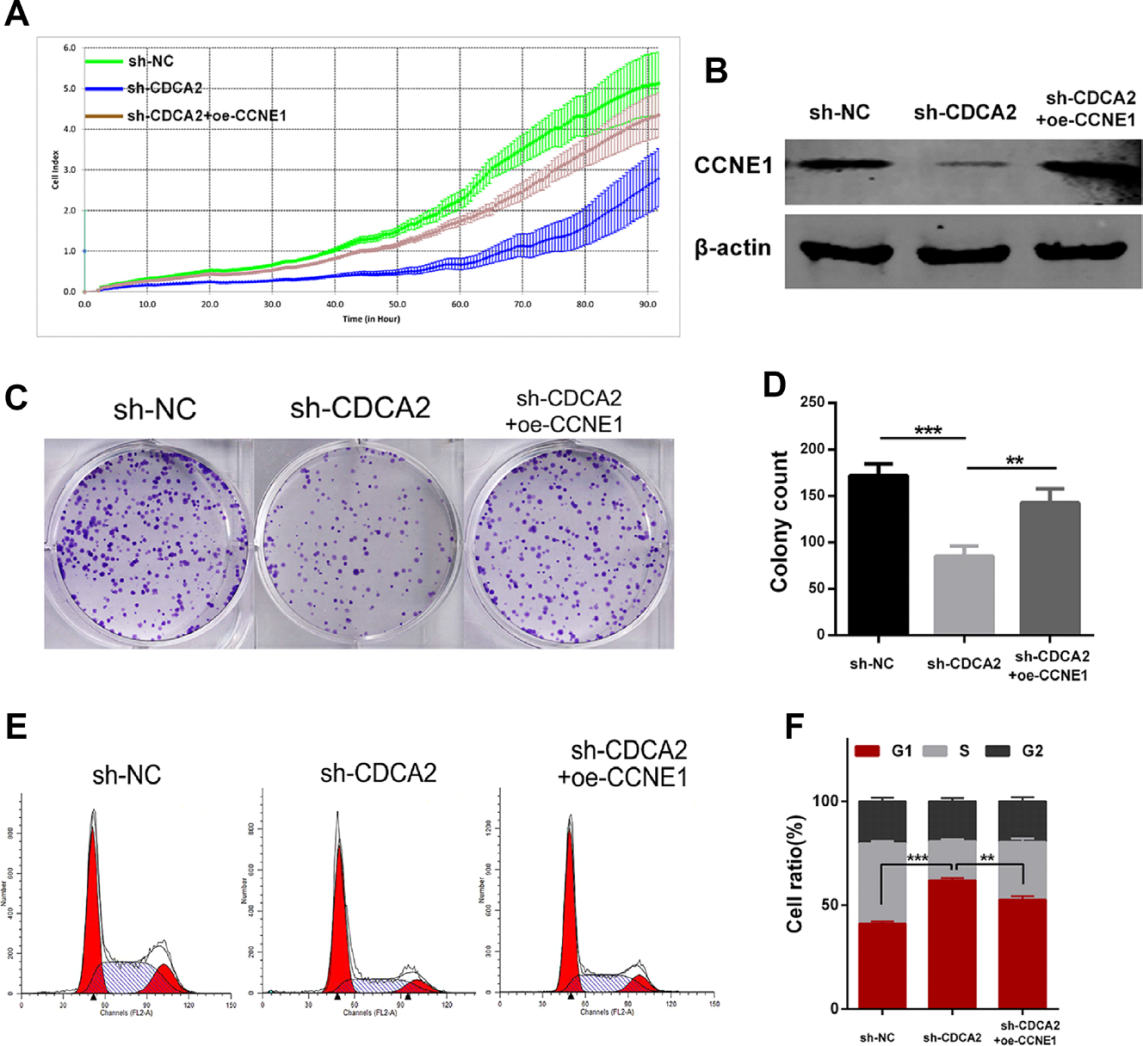
Enforced overexpression of CCNE1 partly rescues the malignant phenotypes in CDCA2-knockdown cells (**A**) Enforced overexpression of CCNE1 (oe-CCNE1) could partially reverse the shRNA-CDCA2-mediated proliferation inhibition of A549 cells. (**B**) Transfection efficiency of CCNE1 was determined by western blot. (**C** and **D**) The colony formation ability in shRNA-CDCA2-mediated A549 cells was partially recovered after enforced oe-CCNE1 treatment (**E** and **F**) Flow-cytometry analysis showed that enforced oe-CCNE1 significantly alleviated shRNA-CDCA2-mediated G1-phase arrest in A549 cells. **p < 0.01, ***p < 0.001.

To examine whether CDCA2 regulated the proliferation of LAC cells via altering CCNE1, sh-CDCA2-treated A549 cells were transfected with pEGFP-N1-CCNE1 plasmid. Transfection efficiency was determined by western blot (Figure 6B). The proliferation ability (Figure 6A) and colony formation abilities (Figure 6C and 6D) in sh-CDCA2 cells were partly recovered after CCNE1 was upregulated. As shown in Figure 6E and 6F, enforced overexpression of CCNE1 (oe-CCNE1) significantly alleviated the sh-CDCA2-mediated arrest in G1 phase (*p* < 0.01).

### Silence of CDCA2 suppressed tumor growth *in vivo*

**Figure 7:**
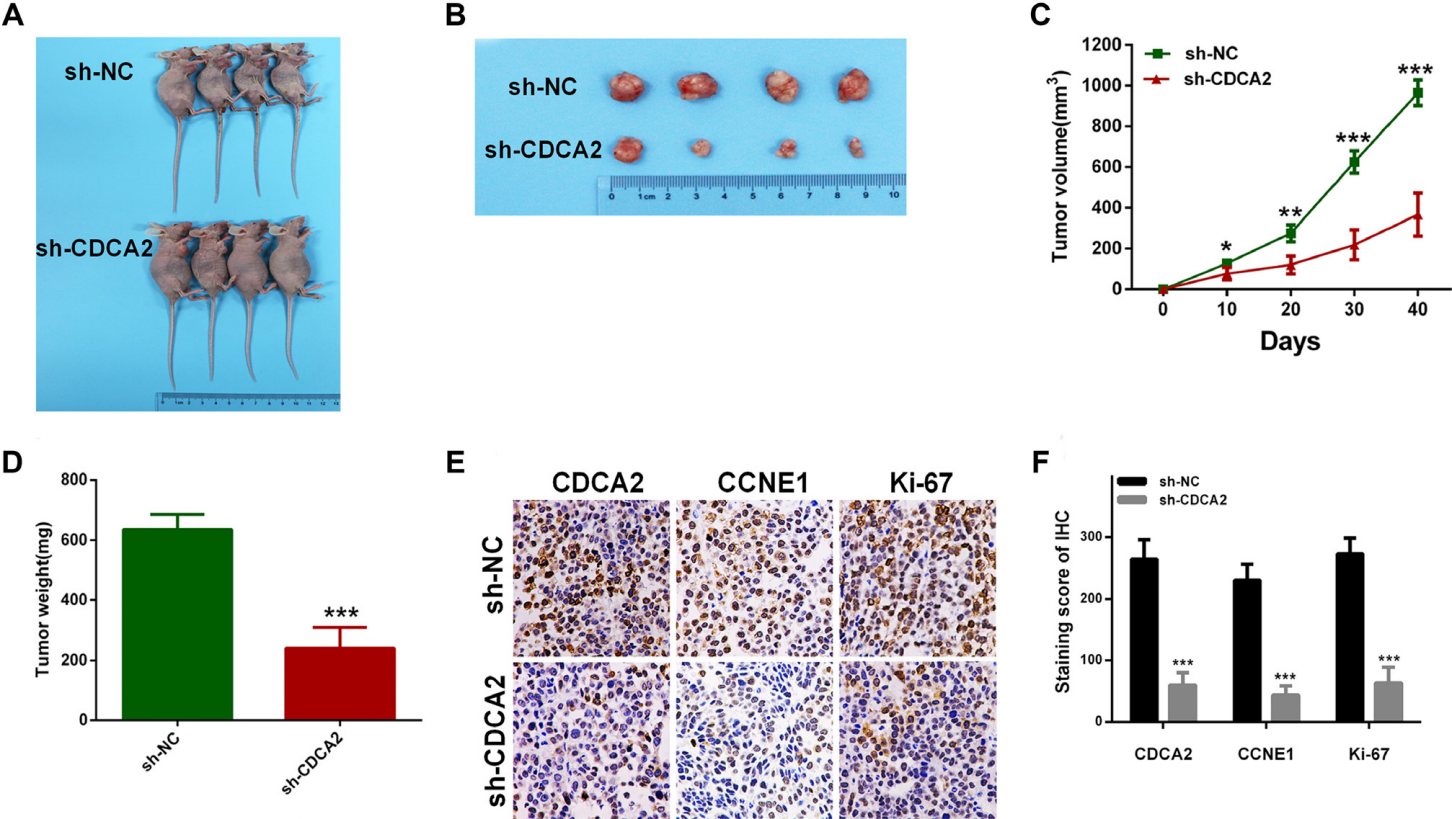
Knockdown of CDCA2 inhibits tumor growth *in vivo* (**A**) Xenograft model in nude mices. (**B**) Nodules harvested from sh-NC group and sh-CDCA2 group. (**C** and **D**) Tumor nodules derived from sh-CDCA2-transfected A549 cells are significantly smaller than those in sh-NC group. (**E** and **F**) Immunohistochemistry showed that CCNE1 and Ki-67 staining was weaker in sh-CDCA2 group, as well as CDCA2.

To assess the oncogenic role of CDCA2 *in vivo*, we established xenograft tumor models using A549 cells transfected with sh-NC or sh-CDCA2. All nude mice developed xenograft tumors at the injection site, and xenograft tumors were harvested 40 days after injection (Figure 7A and 7B). As shown in Figure 7C and 7D, the average tumor volume and weight in the sh-CDCA2 group were significantly lower than those in the sh-NC group. IHC analysis revealed that tumors derived from sh-CDCA2 transfected cells showed weaker staining for Ki-67 and CCNE1 than those in sh-NC group (Figure 7E and 7F).

## DISCUSSION

CDCA2 was first identified as a PP1 binding protein by Laura et al. [12]. Later, Peng et al. reported that the level of CDCA2 determined the activation threshold of the DNA damage checkpoint [4]. DNA damage-induced cell cycle checkpoints transiently delay cell cycle progression in proliferating cells, which may induce cell cycle arrest at specific phases [13, 14]. Vagnarelli et al. also reported that CDCA2 acts as a key regulator in chromatin remodeling by targeting PP1 for the de-phosphorylation of histone H3 [8]. All these studies indicated that CDCA2 plays a critical role in cell cycle progression.

In addition to previous studies that reported CDCA2 upregulation in neuroblastoma [9] and oral squamous cell carcinoma [10], we present the first evidence that CDCA2 upregulation widely occurs in LAC and positively correlates with worse differentiation, greater tumor size and advanced TNM stage in both mRNA and protein levels. Consistent with these clinical findings, experiments on LAC cell lines showed that suppression of CDCA2 significantly inhibited cell proliferation by arresting cell cycle progression at G1 phase, with no effect on apoptosis.

We therefore hypothesized that CDCA2 promotes proliferation by inducing G1/S transition. We therefore measured several G1 phase-related genes to explore the potential mechanism. We found only CCNE1 expression was significantly decreased by siRNA-mediated CDCA2 knockdown, which was consistent with the result of Pearson test between CDCA2 and CCNE1 (*r* = 0.6491, *p* < 0.0001) in LAC tissues in TCGA dataset. Then rescue experiment was performed, and we found that enforced overexpression of CCNE1 could greatly increase proliferative ability and alleviate G1-phase arrest in sh-CDCA2-treated cells. Xenograft assay also showed that depletion of CDCA2 suppressed tumor growth *in vivo* with decreased expression of CCNE1. Cell cycle alteration is one of the hallmarks of cancer [15, 16]. CCNE1 is an important G1 phase-related gene, whose depletion inhibits lung cancer cells proliferation [17]. In addition, limiting the supply of CCNE1 enforces the existence of G1 phase [18]. Furthermore, some studies reported that CCNE1 was upregulated in NSCLC and indicated poor prognosis [19–21].

We also explored the prognostic value of CDCA2. Univariate and multivariate analysis in both TCGA and TMA showed that LAC patients with higher expression of CDCA2 had a worse prognosis than those with lower expression.

In conclusion, our study suggests that CDCA2 is widely upregulated in LAC, and high level of CDCA2 correlates with more advanced tumor stage and worse prognosis. CDCA2 can promote LAC cell proliferation *in vitro* and tumor growth *in vivo*. These findings suggest that CDCA2 plays an oncogenic role in LAC, and CDCA2 might serve as a novel prognostic biomarker in LAC patients.

## MATERIALS AND METHODS

### Bioinformatics analysis

A TCGA dataset named *TCGA_LUAD_exp_HiSeqV2-2015-02-24* was downloaded at the website of the UCSC cancer browser(https://genome-cancer.ucsc.edu/) [22, 23]. The dataset contains a list of 511 LAC samples and among them 57 have paired normal tissue samples. Expression values for CDCA2 were obtained from the “genomicMatrix” file, and all values were normalized. Fisher's paired *t*-test was used to evaluate CDCA2 expression in tumor and para-tumor tissues. Chi-square test and one-way ANOVA were used to analyze the association between clinical characteristics and CDCA2 expression. Kaplan-Meier analysis, log-rank test and Cox regression analysis were used to evaluate the prognostic value of CDCA2 in LAC patients.

Then, a list of 160 genes (details shown in Supplementary Table 3) with highest co-expression correlation (Pearson *r* value > 0.7) with CDCA2 in the TCGA LAC dataset were submitted to Gene Ontology Consortium(http://geneontology.org/) [24, 25] for pathway enrichment analysis.

### Cell lines, cell culture, siRNA and lentivirus-based RNA interference transfection

H1299, H1975 and A549 cells were obtained from American Type Culture Collection (ATCC, USA), while human bronchial epithelial cell (HBE) and SPC-A-1 cells were gifted by Dr. Zhibin Hu. All cells were grown in RPMI1640 media (KeyGEN, Nanjing, China) supplemented with 10% fetal bovine serum and penicillin/streptomycin and cultured at 37°C in a humidified incubator containing 5% CO2. Transfection was performed following the small-interfering RNA (siRNA) sequences transfection protocol for Lipofectamine RNAi MAX (Invitrogen, USA). Nonsense RNAi (nsRNA) was used as a negative control for CDCA2 siRNA. Transfection efficiency was evaluated by quantitative real-time RT-PCR and western blot. Two siRNAs were designed: the sequences were as follows: siRNA-1 for CDCA2: 5′- CACCUGCCUUUCUAAAUAUTT-3′(sense), 5′- AUAUUUAGAAAGGCAGGUGTT-3′(antisense); siRNA-2 for CDCA2: 5′-GGGCAAAGGAUCAAGUG AUTT-3′(sense), 5′-AUCACUUGAUCCUUUGCCCTT-3′(antisense). And the following Nonsense siRNA was used as negative control: 5′- UUCUCCGAACGUG UCACGUTT-3′(sense), 5′-ACGUGACACGUUCGGAGAATT -3′(antisense). The human CDCA2 targeting small hairpin RNA sequence was designed based on siRNA-1 and nsRNA. We generated recombinant lentiviral particles and cells were transfected with CDCA2 or negative control recombinant lentivirus (shRNA-CDCA2 or shRNA-NC, respectively). For overexpressing CDCA2 and CCNE1, CDCA2 cDNA and CCNE1 cDNA were cloned into a pEGFP-N1 vector (purchased from Genechem) to construct overexpression plasmid, and an empty vector (EV) was used as a negative control.

### RNA extraction, reverse transcription and real-time quantitative PCR

Total RNA was extracted from cultured cells using TRIzol reagent (Invitrogen, Carlsbad, CA, USA). For RT-PCR, 1000 ng total RNA was reverse-transcribed to a final volume of 20 μl cDNA using a Reverse Transcription Kit (Takara, cat: RR036A). qRT-PCR analyses were performed with SYBR Select Master Mix (Applied Biosystems, Cat: 4472908). The qRT-PCR primers for CDCA2, p21, p27, CCND1, CCNE1 and β-actin are shown in Supplementary Table 4. The qRT-PCR data collection was performed using a QuantStudioTM 6 Flex Real-Time PCR System and the qRT-PCR reaction included an initial denaturation step at 95°C for 10 min, followed by 40 cycles of 92°C for 15 sec and 60°C for 1 min. Each sample was run in triplicate and the relative expression was calculated and normalized using the 2^−ΔΔCt^ method relative to β-actin.

### Protein preparation and western blot

Cells were harvested and treated with lysis buffer on ice (KeyGEN, Nanjing, China), and a BCA kit (KeyGEN, Nanjing, China) was used to quantify protein concentration. Equal amounts of protein were loaded in SDS–PAGE gels. After separation in the gel, the protein was transferred on a PVDF membrane. Membranes were blocked in 2% BSA in TBS-T for 1 h, and then incubated overnight (4°C) with antibodies against CDCA2 (Abcam, ab209656 1:1000), p21 (santa cruz, sc-397 1:500), p27 (santa cruz, sc-528 1:200), cyclin D1 (CST, 2978 1:1000), cyclin E1 (Abcam, ab7959 1:200) or β-actin (Cell Signaling, 8H10D10 1:1000). After being washed in TBS-T, membranes were incubated with goat anti-rabbit HRP-conjugated secondary antibody (1:10,000; Abcam) or goat anti-mouse HRP-conjugated secondary antibody (1:10,000; Abcam) for 2 h at room temperature. The blots were visualized by ECL detection (Thermo Scientific). All experiments were repeated at least three times independently.

### Cell proliferation assays

The cell proliferation was monitored using a Cell Counting Kit-8(CCK-8) (KeyGEN, Nanjing, China) or the xCELLigence system. For Cell Counting Kit-8, cells were plated in 96-well plates at a density of 2000 cells in 100 μl per well, and the absorbance was measured at 450 nm with an ELx-800 universal microplate reader. Each experiment was repeated independently in quadruplicate. For colony formation assays, a total of 100 transfected cells were placed in a fresh six-well plate and maintained in medium containing 10% FBS; the medium was replaced every 3 or 4 days. After two weeks, cells were fixed with 4% paraformaldehyde and stained with 0.1% crystal violet. Visible colonies were then counted. For each treatment group, wells were assessed in triplicate. For the xCELLigence system, exponentially growing cells with corresponding treatment in complete media were seeded in E-plates at a density of 20,000 per well. The plates were then locked into the RTCA DP device in the incubator. The proliferative ability in each well was automatically monitored by the xCELLigence system and expressed as a “cell index” value. The cell growth was recorded in real-time for 90 h.

### Colony formation assay

For colony formation assay, a total of 100 transfected cells were placed in a fresh six-well plate and maintained in media containing 10% FBS, replacing medium every 3 or 4 days. After two weeks, cells were fixed with 4% paraformaldehyde and stained with 0.1% crystal violet. Visible colonies were counted, and each experiment was repeated three times.

### Flow cytometry analysis

Flow cytometry analysis was performed to detect cell cycle distribution and cell apoptosis. For cell cycle distribution, cells were transferred and fixed in centrifuge tubes containing 4.5 mL of 70% ethanol on ice. The cells were kept in ethanol for at least 2 h at 4°C. Then, the ethanol-suspended cells were centrifuged for 5 min at 300g. Cell pellets were resuspended in 5 mL of PBS for approximately 30s and centrifuged at 300 g for 5 min, then resuspended in 1 mL of PI staining solution and kept in the dark at 37°C for 10 min. Samples were analyzed using a FACSCalibur flow cytometer. The percentage of the cells in G0–G1, S, and G2–M phase were counted and compared. For apoptosis analysis, cells were washed and re-suspended at a concentration of 1 × 10^6^ cells/ml. Then an Annexin V-FITC Apoptosis Detection Kit (BD Biosciences) was used following the manufacturer's protocol. After incubation at room temperature in the dark for 20 min, the cells were immediately analyzed by a FACScan flow cytometer (Becton Dickinson, Franklin Lakes, NJ). All samples were assayed in triplicate.

### Xenograft experiment

All animal studies were conducted in accordance with NIH animal use guidelines and protocols approved by Nanjing Medical University Animal Care Committee. Eight nude mice (ages 4–6 weeks) were purchased from Nanjing Medical University School of Medicine's accredited animal facility. Briefly, 1.0 × 10^6^ exponentially growing A549 cells transfected with shRNA-CDCA2 or shRNA-NC were injected in axilla subcutaneously. Tumor volume was estimated using calipers every ten days as length × width^2^ × 0.5. In the fortieth day after injection, mice were sacrificed, tumor weights were measured and tumors were collected for further analysis.

### TMA and immunohistochemistry

TMA was performed to evaluate the clinical utility of CDCA2 as a prognostic marker. Briefly, formalin-fixed paraffin-embedded archive tissue of 92 paired LAC and adjacent normal lung tissues were arranged in tissue array blocks (Shanghai BioChip Co., Ltd. Shanghai, China). Each spot was accompanied with cases material including sex, age, pathologic grade and clinical stage. The study was in accordance with the provisions of Ethics Committee of Nanjing Medical University. A written informed consent was obtained from each participant involved in this study. This study was approved by the Ethics Boards of the Cancer Institute of Jiangsu Province.

CDCA2 staining was scored independently by two observers (including a pathologist) according to intensity and percentage of positive cells. The staining intensity was scored according to 4 grades: 0 (no staining), 1 (weak staining), 2 (moderate staining), or 3 (intense staining). The product (percentage of positive cells and respective intensity scores) was used as the final staining score (a minimum value of 0 and a maximum of 300).

### Statistical analysis

Student's *t*-test, chi-square test, one-way ANOVA, Cox regression analysis and log-rank test were used to analyze data with SPSS Statistics software (version 20.0, Chicago, Ill). *P* < 0.05 was considered statistically significant.

## SUPPLEMENTARY MATERIALS FIGURES AND TABLES








