# Supplementary material for: CDCA2 promotes lung adenocarcinoma cell proliferation and predicts poor survival in lung adenocarcinoma patients
Source: Oncotarget. 2017 Feb 19;8(12):19768–79. doi: 10.18632/oncotarget.15519 (PMC5386720; doi:10.18632/oncotarget.15519)
Supplement: Supplementary file 1 [file oncotarget-08-19768-s001.pdf]

CDCA2 promotes lung adenocarcinoma cell proliferation and predicts poor survival in lung adenocarcinoma patients

Supplementary Materials

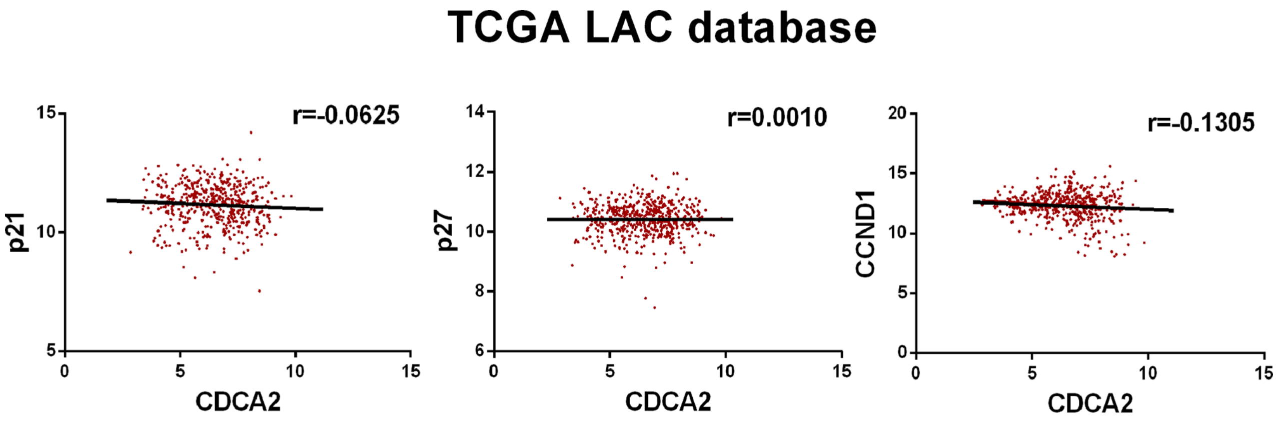

Supplementary Figure 1: Pearson test showed that p21, p27 or CCND1 was not significantly correlated with CDCA2 in TCGA LAC dataset.

Supplementary Table 1: A list of 473 LAC patients with clinical information from TCGA LAC database. See Supplementary_Table_1

Supplementary Table 2: A list of 411 LAC patients with follow-up information from TCGA LAC database. See Supplementary_Table_2

Supplementary Table 3: A list of genes with Pearson correlation values with CDCA2 in TCGA LAC database. See Supplementary_Table_3

Supplementary Table 4: Sequences of qRT-PCR primer

Gene	sense	anti-sense
CDCA2	ATGACCGGCTGTCTGGAAT	GCTGAGACCTTCCTTTCTGGT
CDKN1A	GCAGACCAGCATGACAGATTT	GGATTAGGGCTTCCTCTTGGA
CDKN1B	TGGAGAAGCACTGCAGAGAC	GCGTGTCCTCAGAGTTAGCC
CCND1	GCGCTTCCAACCCACCCTCCATG	GCGCCGCAGGCTTGACTCCAGAA
CCNE1	TTCTTGAGCAACACCCTCTTCTGCAGCC	TCGCCATATACCGGTCAAAGAAATCTTGTGCC
β-actin	GAAATCGTGCGTGACATTAA	AAGGAAGGCTGGAAGAGTG
